# The challenge of Burnout in Public Medical Teachers in Pakistan: A mixed methods study

**DOI:** 10.12669/pjms.37.5.4429

**Published:** 2021

**Authors:** Ali Madeeh Hashmi

**Affiliations:** Ali Madeeh Hashmi MBBS, MD, DABPN, FAPA. Tenured Professor, Department of Psychiatry, King Edward Medical University, Lahore, Pakistan

**Keywords:** Mixed methods, medical teachers, public medical education, burnout

## Abstract

**Background and Objectives:**

Burnout is common in healthcare workers and affects multiple domains of functioning. The objective of this study was to assess burnout in medical teachers in a large public medical university in Lahore, Pakistan and explore the factors behind it.

**Methods:**

Using an explanatory sequential mixed methods design, we first sent out the abbreviated Maslach Burnout Inventory (aMBI) to all teaching faculty of basic and clinical science at King Edward Medical University (KEMU) Lahore. Descriptive analysis was performed on the 203 respondents who returned the survey. Of those who scored higher on the aMBI, 10 respondents (8 clinical science faculty and two basic science faculty) were selected for detailed semi-structured interviews exploring possible reasons for burnout. Thematic analysis of interview transcripts was performed using Interpretive Phenomenological Analysis. Triangulation and member checking was used for validation.

**Results:**

About 38.9% of respondents scored high on the Emotional Exhaustion subscale and 31.5% scored high on the Depersonalization subscale. There were statistically significant differences on the mean Emotional Exhaustion scores (p <0.001) between Basic and Clinical Sciences Departments with respondents from the Clinical Departments having higher scores (7.84 ± 4.32). Four main themes and multiple sub-themes emerged around burnout after qualitative analysis of the data. These included 1. Work-related factors 2. Family and social factors including challenges related specifically to women 3. Feelings and emotions and 4. Personal Qualities.

**Conclusion:**

A significant proportion of medical faculty experiences burnout related to their professional and personal responsibilities. The reasons are varied. Policy planners and University/College administrators must acknowledge the negative effects of burnout on medical teachers and take steps to ameliorate it in the interests of improving medical education and training.

## INTRODUCTION

***Canst thou not minister to a mind diseased***,

***Pluck from the memory a rooted sorrow***,

***Raze out the written troubles of the brain***

***And with some sweet oblivious antidote***

***Cleanse the stuff’d bosom of that perilous stuff***

***Which weighs upon the heart?*** - *Shakespeare-MacBeth.^1^*

‘Burnout’ was first reported in the 1970s by American Psychologist Herbert Freudenberger who initially used the term to describe a constellation of symptoms found within the ‘helping professions’; in his case, doctors and nurses.^2^ American Psychologist Christina Maslach and others developed one of the earliest tools for measuring burnout, the Maslach Burnout Inventory.^3^ Since their ground breaking work, the concept of burnout has been extended beyond the healthcare professions and is now known to affect not just doctors and nurses but everyone from busy professionals to home makers.

There is now a significant amount of published literature on this phenomenon including in Pakistan.^4^-^6^ but no researcher, to our knowledge, has attempted an in-depth exploration of the phenomenon of burnout in medical teachers especially in the public setting.

Unsurprisingly, given the challenges facing doctors and healthcare professionals in a low and middle income country like Pakistan, most previous studies reported a high prevalence of burnout ranging between 35% to 80% in doctors and nurses. A large number of studies have documented the negative effects of burnout in healthcare professionals^7^. These studies have reported that burnout can degrade professionalism, negatively influence the quality of care delivered to patients, increase risk for medical errors and induce physicians and healthcare professionals to leave the profession.^7^

Much has been written about the problems of medical education in Pakistan.^8^,^9^ These challenges to medical education are even more urgent in public medical sector teaching institutions given the larger medical student class sizes as well as the huge number of patients being treated in public hospitals in Pakistan.

Given the relative lack of data on burnout in medical teachers, especially in the public medical setting, the rationale of our study was to explore burnout in medical teachers so policy planners in government, heads of medical teaching institutions and administrators can implement appropriate remedial measures.

## METHODS

In this explanatory sequential mixed-methods study conducted in the pragmatic paradigm, we wanted to explore the phenomenon of burnout in medical teachers at King Edward Medical University (KEMU), the oldest public institution of medical learning in the country using Interpretive Phenomenological Analysis (IPA). We also wanted to assess whether the experience of burnout differed among Basic Science versus Clinical Science faculty and the possible factors contributing to burnout.

In the initial, quantitative phase of our study, 203 medical teachers at KEMU were selected on the basis of non-probability consecutive sampling. We calculated that a sample size of 264 faculty members would be adequate using 5% level of significance, 80% power of test with expected proportions of burnout cases as 39% and 56% in basics (Pathology) and clinical specialties (Internal medicine) respectively^10^. A response rate of 60-70% is generally considered satisfactory for quantitative surveys^11^. 203 faculty members responded to our survey. This 76.8% response rate was adequate for our purposes.

After informed consent, those who volunteered to participate were briefed about the objectives of the study, and confidentiality of responses was ensured by maintaining anonymity of responders. All the faculty members who agreed to participate were administered the abbreviated Maslach Burnout Inventory (aMBI) to assess whether they were experiencing burnout and to what extent. They were also asked some additional questions which could possibly contribute to burnout such as age, gender, marital status, number of children, commute/travel time from home to work and back, whether they were doing private practice in addition to their teaching job, their department and rank.

The original MBI^3^ also referred to as the Maslach Burnout Inventory-Human Services Survey (MBI-HSS) is a 22 question survey with three subscales to measure the different domains of burnout: Emotional Exhaustion (EE), depersonalization (DP) and a sense of low Personal Achievement (PA). We used an abbreviated version of the MBI (aMBI) which has been validated in practice^12^. It consists of three item versions of the same subscales. Data analysis was done via IBM SPSS 23. Descriptive findings were recorded as frequencies and percentages for background characteristics. The overall Burnout Scores and Dimensional scores of Emotional Exhaustion and Depersonalization were recorded as Mean and Standard Deviation. Inferential statistical analysis was done via Pearson’s Chi-square test and Independent t-test. The comparison of mean scores between the faculty members from Basic and Clinical Sciences Departments was done by applying Independent sample t-test with p < 0.05. Emotional Exhaustion and Depersonalization were recoded and dichotomized into Low and High Scores with Emotional Exhaustion Scores ≥9 and Depersonalization score ≥6 regarded as the high scores, the same cutoff scores that were used to interview the participants for the qualitative section. Pearson’s chi-square test was used to determine the distribution of high and low dimensional scores across different categories of background characteristics such as age, gender, marital status, number of children, home to work commute distance in minutes, private job or practice and professional rank in the department.

In the second, qualitative phase , ten faculty members (two Basic Science Faculty and eight Clinical Science Faculty) were purposively selected for semi-structured, in-depth interviews to explore, in detail, the reasons for burnout. These faculty members were the ones who had scored higher on the burnout survey. We included different ranks (Professors, Associate/Assistant Professors, Senior Registrars, Demonstrators) as well as different specialties (Clinical and Basic Sciences) to make the responses more representative. Participants were briefed about the objectives of the study and, after informed consent, the interviews were audio recorded. Data saturation was achieved after eight interviews and further two interviews were conducted to confirm saturation. The interviews were transcribed and thematic analysis was done for data reduction and interpretation. Validity of the data was ensured by triangulation and member checking and verbatim comments are given for confirmation.

### Ethical Approval

(Ref: 137/RC/KEMU, Dated: 14-02-2018).

## RESULTS

The back ground characteristics of the respondents are shown in Table-I. The descriptive findings for Burnout Dimensions are shown in Table-II.

**Table-I:** Background characteristics of the study participants.

Background Characteristics	Frequency *(f)*	Percentage *(%)*
***Age***		
25-35 years	86	42.4%
36-45 years	75	36.9%
46-60 years	42	20.7%
Gender		
Male	84	41.4%
Female	119	58.6%
Marital Status		
Single	24	11.8%
Married	179	88.2%
Number of Children		
No children	36	17.7%
1-3 children	156	76.8%
4 and > 4 children	11	5.4%
***Distance from home to workplace in minutes***		
≤ 30 minutes	95	46.8%
31-60 minutes	103	50.7%
> 60 minutes	5	2.5%
***Basic Sciences or Clinical Department***		
Basic Sciences Department	68	33.5%
Clinical Department	134	66%
Missing	1	0.5%
***Private Practice/ Job***		
Yes	71	35%
No	126	62.1%
Not Applicable	6	3%
***Department***		
Anatomy	28	13.8%
Physiology	10	4.9%
Biochemistry	10	4.9%
Pharmacology	8	3.9%
Forensic Medicine and	5	2.5%
Toxicology	1	0.5%
Pathology	6	3%
Community Medicine	2	1%
Anesthesia	33	16.3%
Medicine and Allied	37	18.2%
Surgery and Allied	5	2.5%
Pediatrics	41	20.2%
Obstetrics and Gynecology	5	2.5%
Psychiatry	11	5.4%
Ophthalmology	1	0.5%
Missing		
***Current Rank in Department***		
Professor	20	9.9%
Associate Professor	14	6.9%
Assistant Professor	55	27.1%
Senior Registrar	52	25.6%
Demonstrator	62	30.5%

Total	203	100%

**Table-II:** Descriptive findings for Burnout subscale dimensions.

Burnout Subscale/ dimension	*f*	*%*	Mean ± SD	95% C.I for Mean		Median	IQR	Min	Max	Range

				Lower Bound	Upper Bound					
***Emotional Exhaustion***										
High Scores ≥ 9	79	38.9%	6.86 ± 0.33	6.2	7.5	6	7	0	18	18
Low Scores < 9	124	61.1%								
***De-personalization***										
High Scores ≥ 6	64	31.5%	3.6 ± 0.29	3	4.1	2.5	6	0	18	18
Low Scores < 6	139	68.5%								
Overall Burnout Score			10.46 ± 0.53	9.4	11.5	9	10	0	36	36

The mean score for Emotional Exhaustion was 6.86 ± 0.33 and for Depersonalization was 3.6 ± 0.29 as demonstrated in Table-II. The overall Burnout Score was recorded as 10.46 ± 0.53. The mean Emotional Exhaustion scores of ≥ 9 and the mean Depersonalization scores of ≥ 6 were considered as high.

The breakdown of the two burnout scales across the background characteristics of the respondents is shown in Tables-III and IV.

**Table-III:** Emotional Exhaustion across background characteristics.

Background Characteristics	Mean ± SD				*p*

Basic Sciences Departments Clinical Sciences Departments	5.5 ± 4.48 7.84 ± 4.32				<0.001

	High Emotional Exhaustion		Low Emotional Exhaustion		

	*f*	*%*	*f*	*%*	
***Age***					0.8
25-35 years	33	38.4%	53	61.6%	
36-45 years	31	41.3%	44	58.7%	
46-60 years	15	35.7%	27	64.3%	
***Gender***					0.1
Male	28	33.3%	56	66.7%	
Female	51	42.9%	68	57.1%	
***Marital Status***					0.1
Single	6	25%	18	75%	
Married	73	40.8%	106	59.2%	
***Number of Children***					0.5
No children	11	30.6%	25	69.4%	
1-3 children	64	41%	92	59%	
4 and > 4 children	4	36.4%	7	63.6%	
***Distance from home to workplace in minutes***					0.6
≤ 30 minutes	36	37.9%	59	62.1%	
31-60 minutes	42	40.8%	61	59.2%	
> 60 minutes	1	20%	4	80%	
***Basic Sciences or Clinical Department***					0.001
Basic Sciences Department			53	77.9%	
Clinical Department	15	22.1%	70	52.2%	
Missing	64	47.8%	1	100%	
***Private Practice/ Job***					0.2
Yes	32	45.1%	39	54.9%	
No	46	36.5%	80	63.5%	
Not Applicable	1	16.7%	5	83.3%	
***Current Rank in Department***					0.001
Professor	7	35%	13	65%	
Associate Professor	6	42.9%	8	57.1%	
Assistant Professor	20	36.4%	35	63.6%	
Senior Registrar	32	61.5%	20	38.5%	
Demonstrator	14	22.6%	48	77.4%

High Emotional Exhaustion= Mean Emotional Exhaustion score ≥9; p <0.05.

**Table-IV:** Depersonalization across background characteristics.

Background Characteristics	Mean ± SD				*p*

Basic Sciences Departments Clinical Sciences Departments	3.76 ± 4.3 3.63 ± 3.5				0.8

	High Depersonalization		Low Depersonalization		

	*f*	*%*	*f*	*%*	
***Age***					0.02
25-35 years	30	34.9%	56	65.1%	
36-45 years	28	37.3%	47	62.7%	
46-60 years	6	14.3%	36	85.7%	
***Gender***					0.6
Male	28	33.3%	56	66.7%	
Female	36	30.3%	83	69.7%	
***Marital Status***					0.5
Single	9	37.5%	15	62.5%	
Married	55	30.7%	124	69.3%	
***Number of Children***					0.8
No children	10	27.8%	26	72.2%	
1-3 children	51	32.7%	105	67.3%	
4 and > 4 children	3	27.3%	8	72.7%	
***Distance from home to workplace in minutes***					0.4
≤ 30 minutes	34	35.8%	61	64.2%	
31-60 minutes	29	28.2%	74	71.8%	
> 60 minutes	1	20%	4	80%	
***Basic Sciences or Clinical Department***					0.7
Basic Sciences Department			45	66.2%	
Clinical Department	23	33.8%	93	69.4%	
Missing	41	30.6%	1	100%	
***Private Practice/ Job***					0.8
Yes	24	33.8%	47	66.2%	
No	38	30.2%	88	69.8%	
Not Applicable	2	33.3%	4	66.7%	
***Current Rank in Department***					0.05
Professor	1	5%	19	95%	
Associate Professor	6	42.9%	8	57.1%	
Assistant Professor	16	29.1%	39	70.9%	
Senior Registrar	21	40.4%	31	59.6%	
Demonstrator	20	32.3%	42	67.7%	

High Depersonalization= Mean Depersonalization scores ≥6; *p* <0.05.

For the qualitative part of our study, a total of 10 medical teachers were interviewed via semi-structured interviews using the questions on the aMBI as starting points. The respondents included one senior Professor of Surgery (R-1), a senior Professor of Medicine (R-2), a Professor of Anesthesia (R-3), an Associate Professor of Medicine (Head of Department of one of the medical units-R-4), an Assistant Professor of Obstetrics/Gynecology (R-5), an Assistant Professor of Orthopedic Surgery (R-6), a Demonstrator (Lecturer) in Community Medicine/Public Health (R-7), a Demonstrator in Biochemistry (R-8), an Assistant Professor in Emergency Medicine (R-9) and a Senior Registrar in Surgery (R-10).

We performed qualitative thematic analysis of our data using Interpretive Phenomenological Analysis (IPA), a method widely used in qualitative research which is particularly suited to explore the ‘lived experiences’ of participants of the phenomenon in question.^13^

Four main themes related to burnout emerged from the participant interviews: Work related factors, Family or social factors, Feelings/Emotions and Personal Qualities. Sub-themes (detailed below) generally fell along the following lines: those that contributed to increasing and worsening burnout included issues like work load, teaching, multi-tasking, women’s challenges, social life outside work, uncertainty and self-doubt, etc. There were, interestingly also some sub-themes that could be considered ‘anti-burnout’ such as gratitude, mastery, passion, focus, meticulousness, personal improvement and personal accomplishment. The major themes, sub-themes, illustrative categories and representative quotes are as below:

***Theme-1:*** Work-Related Factors

***Sub-Theme-1:*** High Responsibility

***Category:*** Personal responsibility

*“(My teacher) used to say ‘if instead of the patient your father or brother was on the bed, would you have checked them the same way?’ I still remember this phrase. And because of this, I take every patient very personally. My parents say ‘you die along with the patient who dies’ so that shows my level of involvement” (R-4)*.

***Category:*** High Acuity of patients

*“(Our Hospital) is a tertiary care hospital which has been known over the decades for its maternity care…most of the exacerbated cases from the periphery come here (in very bad shape)- I usually get called for patients who have collapsed” (R-5)*.

Sub-Theme 2: Work Environment

***Category:*** High Work Load

*“Enormity of work load here is greater than other institutions. It is because this institution is a very big university and encompasses a lot of work and departments unlike anywhere else” R-1*

***Category:*** Work pressure, thoughts of leaving medical field.

*“There are times, like in COVID days, we came across scenarios with high mortality, helplessness, not enough medication, resources, or knowledge. Sometimes I think I (should) have opted for something else other than medicine” R-2*.

***Category:*** Work Schedule

*“(I am on call ) on Alternate days! I have been awake for 24 hours now and today is supposed to be my free day. The same cycle is going to repeat from tomorrow. It’s ongoing” R-3*

***Category:*** (Lack of) Job Description, Workplace politics

*“There isn’t any set scale and specifications of job, neither there is any yard stick for achievement. We are handling our jobs and students in pretty random way. There is unequal distribution (of work). Everyone will try to shun away their work to the next person” R-7*

Category: Work Conditions, Work Load

*“Sometimes, we get short on staff. We are only five permanent demonstrators and we have to take classes, practical and tutorials of all sessions. We are caught up in so many minute nonacademic things in department plus the pressures of personal and family life that we never get any protected time to do and work on our research projects. R-8*

***Theme 2:***

*Family and Social Factors*

***Sub-Theme 1:*** Women’s Challenges

Category: Family Responsibility

*“The schedule and workload at this hospital is quite hectic. I have two sons one in A levels and the other in O levels who are always on my mind, and also my parents live near me who are also getting quite old. And of course my father in law is also in close vicinity and these are some relations that have to be tactfully dealt with. Then there are 4 -5 servants who I have to manage. Then in the evening I go to (private practice) around 6pm which is a world of its own. So naturally I would feel tired”. R-5*

***Sub-Theme-2:*** Family and Social Issues

***Category:*** Family and Social Bias

*“Even though (my family) are very educated, but still they have a bias against teaching in basic sciences*. *The main frustration is that no one considers you a doctor. You are doing a lot, but still no one considers you anything” R-8*

***Category:*** Social Life outside Work

*“I have (few) friends outside of work, or social backup outside of my professional life. Here, in my experience, friendship at the professional level stays that way. Once you get transferred or the other person gets transferred, you stop being friends in that sense. So obviously if you are spending most of your time at work, you have a good chance at making friends at work too but I am not good at that. R-4*

***Theme-3:***

*Feelings/Emotions*

***Sub-Theme 1:*** Negative Feelings

***Category:*** Fatigue (Physical and Mental)

*“Things get so overburdened. You treat the first patient with complete focus and concentration, but when you reach the tenth or twelfth patient, you cannot do it anymore” R -10*

***Categories:*** Uncertainty/Self-Doubt, Regret

*“Of course these thoughts come when you feel burned out and frustrated. I quite frequently feel like I shouldn’t have opted for anesthesia and even medicine sometimes” R-3*

***Sub-Theme 2:*** Positive Feelings

***Categories:*** Gratitude/Thankfulness, Equanimity, Mastery

*“When your students old or new, or trainees contact you, you feel that you had a positive influence. Especially with your patients. The most satisfaction comes from them. These occasions make me feel good” R-1*

***Theme-4:*** Personal Qualities, Habits and Preferences

***Sub-Theme 1:*** Positive

***Categories:*** Focus, meticulousness, Personal Improvement/Accomplishment

*“If I look at the professional aspect, my residents and colleagues are always impressed by me because I check the patient carefully and I perform my duties well and they learn from whatever I have taught them. They feel that after working with me, they learn better” R-4*

***Sub-Theme 2:*** Negative

***Categories:*** Personal Health, habits, appearance, privacy and ‘personal distance’.

*“The interaction with people apart from patients which I would term as “chatter”, is what I usually want to avoid. This includes people getting in my personal domain and asking personal questions. I don’t like that kind of interaction” R-2*.

## DISCUSSION

Multiple studies have documented the high rates of burnout in healthcare professionals.^4^-^6^ but none, to our knowledge have attempted an in-depth exploration of this phenomenon especially in medical teachers in the public setting. This is important since medical institutions are the ‘petri dishes’ which are cultivating, grooming and training the next generation of healthcare professionals. If medical teachers are experiencing high rates of burnout, this will inevitably affect their morale and work efficiency and can even lead to grievous medical errors or induce a medical teacher to leave the medical field entirely.^7^

Multiple studies have documented the critical shortage of physicians in lower and middle income countries including Pakistan for a number of reasons including emigration.^14^ Countries like Pakistan can ill-afford to lose medical teachers who are already in short supply especially in public medical colleges and universities where medical student class sizes can often exceed 300 students per class. In addition, these medical teachers are also the primary resource for teaching and training post-graduate residents and fellows.

Our study confirmed previous findings by multiple researchers of the high rates of burnout in healthcare workers.^4^-^6^ In our study 38.9% of the medical teachers surveyed showed high burnout scores on the ‘EE’ subscale of the aMBI while 31.5% showed high burnout scores on the ‘DP’ subscale. When we interviewed some of these medical teachers, the major themes related to burnout were as expected: high work load, lack of time for leisure, recreation and family matters, family and social attitudes towards their work (especially in women) and personal feelings of inadequacy, regret, mental and physical fatigue and self-doubt. In some cases, these feelings were balanced by feelings of gratitude (at being able to help people), mastery of new skills, passion for their work and equanimity, a feeling best described as calmness and composure in the face of difficulty situations.

Some personal habits appeared to contribute positively towards medical teachers’ ability to combat burnout such as being meticulous in their daily duties (which increased feelings of mastery), bringing complete focus to their work (which helped them block out other matters) and a sense of personal improvement and accomplishment. Other personal habits appeared to detract from equanimity and increase feelings of burnout such as inquiries from others about a teacher’s personal health or appearance, their own daily habits or the requirement to interact socially with family and friends following a long, grueling day at work.

It is important to note that three of the four major themes that emerged from the qualitative analysis of our data were related to what might be called ‘modifiable’ factors. For instance, some “Work-related Factors” can be improved by simple interventions such as developing written job descriptions of teachers, distributing work load more equitably and providing opportunities for professional advancement. The two themes related to medical teachers themselves, “Feelings/Emotions” and “Personal Qualities” can also be addressed effectively via training workshops related to both medical education and mental health.

### Strengths

The strengths of our study include the large cross section of medical teachers who we included in our initial sample. Our respondents were quite open about the reasons for their burnout which allowed us to explore this in detail.

### Limitations

One limitation of our study is the smaller number of Basic Science teachers who responded to our initial survey (partly because they are a smaller proportion of the faculty at KEMU). Thus the views of Basic Science teachers may be under-represented. Future studies should target this group more effectively since they are instrumental in the initial years of undergraduate medical education. Our study was limited to one public medical university and the results cannot be generalized to other public medical colleges and universities.

## CONCLUSION

Burnout is widespread in the medical fraternity, including medical teachers, and can be debilitating. Public medical teachers are already in short supply in Pakistan and if they suffer burnout or, in extreme cases, leave the profession, it will strain the medical education system further. There are many factors contributing to burnout in medical teachers which can be fixed with relatively simple remedial measures. Addressing burnout in medical teachers will improve their efficiency, prolong their careers and thus improve medical education in Pakistan. Medical policy makers, health bureaucrats, University Vice-Chancellors, Principals and Administrators need to pay attention to ameliorating burnout if they want to improve the medical education system of Pakistan.
